# CheMLT-F: multitask learning in biochemistry through transformer fusion

**DOI:** 10.1186/s13321-026-01199-1

**Published:** 2026-04-16

**Authors:** Vladislav Mun, Siamac Fazli

**Affiliations:** Department of Computer Science, Nazarbayev University, Kabanbay Batyr Ave 53, 010000 Astana, Kazakhstan

**Keywords:** Multitask learning, Transformers, Toxicity prediction, Physicochemical properties, Drug–target affinity, Drug screening

## Abstract

Drug discovery remains a slow and costly process, in part because efficacy, toxicity, and physicochemical liabilities must be screened across a vast chemical space. Stand-alone, single-task predictors can help, but they lead to fragmented workflows and make it hard to reuse learned representations, data processing, and infrastructure across endpoints (i.e., prediction tasks). Here we present *CheMLT-F*, a compact multitask transformer that fuses encoders for molecular and protein sequences to learn a unified representation spanning *680+ endpoints*, including diverse toxicities, physicochemical properties, and drug–target interactions. Across 13 public benchmarks, CheMLT-F matches state-of-the-art toxicity classifiers and sets new performance marks for physicochemical property prediction, while remaining competitive for drug–target affinity (KIBA and Davis). Moreover, CheMLT-F demonstrates competitive performance on an external protein-family benchmark spanning seven target superfamilies, indicating broad generalizability in bioactivity prediction. Multitask parameter sharing keeps the model lightweight and inference-efficient, and its modular heads make extensions to new endpoints straightforward. By replacing many individual models with a single, extensible backbone, CheMLT-F streamlines in silico screening and lowers the barrier to broad, data-driven decision-making in early drug discovery.

**Scientific contribution**

We introduce a unified transformer architecture that jointly models molecular and protein sequences across hundreds of pharmacologically relevant endpoints spanning toxicity, physicochemical properties, and drug–target interactions. A tailored training strategy that combines partial encoder freezing, global–local loss balancing, and weighted task sampling reduces trainable parameters and deployment complexity while preserving strong cross-domain generalization. Comprehensive evaluation across 13 public datasets, including scaffold-aware and random data splits, demonstrates competitive accuracy with substantially lower operational overhead than maintaining numerous single-task models, establishing a scalable foundation for extensible and holistic predictive modeling in computational drug discovery.

## Introduction

Drug discovery is a high-risk and capital-intensive process: bringing a single new molecular entity to market typically spans a decade and is often quoted at $2–3B in capitalized R&D costs, with substantial dispersion across modalities and disease areas [1, 2]. Despite process innovations, overall productivity has trended downward (“Eroom’s law”) as regulatory expectations for safety rise and biological complexity increases [3]. The challenge is amplified by the vastness of small-molecule chemical space: for drug-like molecules ($$<500$$Da), the number of synthetically accessible structures is commonly estimated at $$\sim 10^{30}$$-$$10^{60}$$, rendering exhaustive physical screens infeasible [4–6]. To limit late-stage failures, early discovery must prioritize compounds by efficacy (biological target engagement, binding affinity) and safety/ADMET risks [7, 8]. These constraints motivate automated data-driven models that can reduce the 9-year drug development time without additional regulatory interventions [9, 10].

To address these challenges, machine learning (ML) and deep learning (DL) models have emerged as powerful tools for predicting drug physicochemical properties [11–14], drug-target interactions [15–20] and different types of toxicities [11–14, 21]. Although conventional ML and DL approaches may provide reasonable results [17, 21, 22], the most recent state-of-the-art approaches often employ more advanced methodologies, such as transformers [11, 12, 18–20, 23, 24], graph neural networks (GNNs) [13–16, 25, 26] and their combinations [27]. Rather than extracting predefined features (molecular fingerprints [28]), transformer models learn directly from Simplified Molecular Input Line Entry System (SMILES) strings via attention-based learning. GNNs, on the other hand, rely on additional positional and structural information to capture atomic interactions and representations more effectively. More broadly, large-scale multi-task QSAR efforts such as MELLODDY have demonstrated the value of jointly modeling ADME, physicochemical, and bioassay endpoints across industrial-scale datasets using ECFP6 fingerprints and SparseChem feedforward networks [29]. Importantly, MELLODDY addresses a federated QSAR setting and does not incorporate explicit target-sequence modeling or explore transformer- or GNN-based architectures in that framework [29]. This leaves open a complementary question: whether a single unified architecture can combine modern sequence-based molecular modeling with target-aware drug–target interaction prediction while simultaneously supporting molecule-only endpoints on public benchmarks.

Large repositories such as ZINC, ChEMBL, and PubChem [30–32] and curated benchmarks like MoleculeNet/DeepChem [22, 33] have enabled data-driven screening at scale, while repurposing libraries (e.g., the Drug Repurposing Hub [34]) offer smaller, practical search spaces (e.g., the discovery of the antibiotic halicin via data-driven screening [35]). Still, effective prioritization depends on modeling target-specific activity and toxicity: despite valuable DTI resources (e.g., BindingDB, KIBA, Davis), coverage remains sparse and assays are heterogeneous. Repositories of precomputed predictions (e.g., DTA Atlas [36]) alleviate some computational costs but cannot scale to the vast small-molecule universe, require continuous upkeep, and still fragment workflows across models and endpoints. Taken together, these resources form a complex and rapidly expanding ecosystem of datasets, predictive models, and analysis tools. Recent surveys of computational drug repurposing highlight the scale and diversity of these in silico resources and their role in selecting candidate molecules and therapeutic hypotheses [37]. Such heterogeneity in task definitions and measurement protocols across datasets complicates fair evaluation and generalization, making scaffold-aware splits essential for assessing model performance on previously unseen chemical structures [22]. From an application standpoint, real-world discovery workflows benefit from models that are compact and easily extensible. Methodologically, multitask learning (MTL) can mitigate label sparsity and class imbalance by sharing representations across related endpoints, improving data efficiency and generalization [38, 39].

To this end, we introduce CheMLT-F, a unified transformer-based model that predicts 683 endpoints spanning toxicities, physicochemical properties, and drug–target interactions. In contrast to prior multi-task QSAR frameworks such as MELLODDY, CheMLT-F is designed to unify molecule-only endpoints and target-aware DTI affinity prediction on public benchmarks. The model learns end-to-end from both SMILES and protein sequences within a shared transformer backbone. This multitask design enables CheMLT-F to learn shared representations across a wide range of drug-relevant tasks. To stabilize learning across heterogeneous benchmarks, we employ a dataset-aware training strategy that combines task-appropriate local losses with global reweighting. By leveraging shared parameters, the model remains efficient, easy to use, and competitive with state-of-the-art methods. The architecture is readily extensible to additional tasks and provides a practical, general-purpose interface for rapid in silico screening.

## Methods

### Datasets

In this study, we utilized 13 publicly available datasets (see Table 1 for an overview). These datasets can be grouped into three main categories: (i) toxicity/bioactivity classification; (ii) physicochemical property prediction; and (iii) drug–target binding affinity (DTI) regression. With the exception of KIBA and Davis, all datasets were obtained via the DeepChem framework [33] and are also indexed and standardized in Therapeutics Data Commons (TDC) [40, 41]. All datasets were pre-processed with RDKit [42]. Unless stated otherwise, we evaluate our proposed models under both randomized and scaffold-aware splits to assess generalization [22]. In addition to these molecular benchmarks, we also evaluate our approach on an external benchmark consisting of seven protein superfamilies commonly used in drug–target interaction studies [18, 43].

**Table 1:** Summary of datasets used for molecular property prediction, bioactivity modeling, and DTI

Dataset	Category	Task type	Labels	Entries
ToxCast	Toxicity/bioactivity	Classification	617	8,577
SIDER	Toxicity/bioactivity	Classification	27	1,427
MUV	Toxicity/bioactivity	Classification	17	93,087
Tox21	Toxicity/bioactivity	Classification	12	7,831
ClinTox	Toxicity/bioactivity	Classification	2	1,480
HIV	Toxicity/bioactivity	Classification	1	41,127
BACE	Toxicity/Bioactivity	Classification	1	1,513
BBBP	Physicochemical	Classification	1	2,039
Lipo	Physicochemical	Regression	1	4,200
Delaney (ESOL)	Physicochemical	Regression	1	1,128
FreeSolv	Physicochemical	Regression	1	642
KIBA	Binding affinity	Regression	1	118,254
Davis	Binding affinity	Regression	1	30,056
Total			683	311,361

“Labels” denotes the number of endpoints/tasks; “Entries” denotes the number of molecules or molecule–target pairs

#### Toxicity and bioactivity

Toxicity and bioactivity prediction is crucial in drug discovery to identify potential adverse effects before initiation of clinical trials. We employ the following frequently used benchmark datasets to train machine learning models for toxicity classification:

- **ToxCast** contains 8577 compounds with 617 assay endpoints relevant to predictive toxicology, including inhibition of common nuclear receptors and pathways (e.g., androgen receptor, estrogen receptor, PPAR).
- **SIDER** focuses on adverse drug reactions (ADRs), linking 1427 marketed drugs to 27 types of side effects, such as gastrointestinal, nervous system, skin and subcutaneous tissue, cardiac, and hepatobiliary disorders.
- **MUV** (Maximum Unbiased Validation) comprises 93,087 molecules and 17 *binary* classification tasks predicting assay activity (active vs. inactive/decoy) across diverse PubChem bioassays; actives/decoys are curated to minimize analogue bias.
- **Tox21** is a well-known dataset derived from the Tox21 research initiative, containing 7831 compounds with 12 toxicity labels representing distinct biological pathways.
- **ClinTox** contains data on clinically tested drugs, distinguishing between those that were approved and those that failed due to toxicity. It consists of 1480 compounds with two binary labels representing FDA approval and clinical toxicity outcomes.
- **HIV** includes 41,127 molecules labeled as active or inactive for inhibition of HIV replication.
- **BACE** ($\beta$-secretase 1) provides 1513 compounds for classification of BACE1 inhibition, which is relevant in Alzheimer’s disease research.

All molecule-only classification datasets in this study are treated as compound-to-label tasks, consistent with standard benchmark usage. This includes datasets such as BACE, which corresponds to a single fixed target (BACE1) but is distributed without varying protein-sequence inputs across samples. Representing such datasets in a DTI-style format would therefore not introduce sample-specific protein information and would be inconsistent with their standard evaluation protocol.

#### Physicochemical properties

Physicochemical properties of molecules influence their solubility, lipophilicity, and permeability, which are key factors of ADME/ADMET. We utilize the following datasets:

- **BBBP** (Blood-Brain Barrier Permeability) contains 2039 molecules labeled based on their ability to cross the blood-brain barrier (BBB), a key factor in central nervous system (CNS) drug design.
- **Lipo** (Lipophilicity) includes 4200 compounds with experimentally measured logD values, a critical property for drug absorption and distribution.
- **Delaney/ESOL** (Aqueous Solubility): This dataset contains 1128 compounds with experimentally measured solubility values (logS), important for predicting drug dissolution.
- **FreeSolv** provides hydration free energy values for 642 compounds, which are useful in solubility and permeability modeling.

#### Binding affinity

Binding-affinity datasets capture small-molecule interactions with proteins, critical for drug–target discovery. Two prominent datasets include:

- **KIBA** is a large-scale kinase inhibitor dataset with 118,254 drug-target interaction records. It provides KIBA scores, a hybrid metric that combines $K_i$, $K_d$, and $\textrm{IC}_{50}$.
- **Davis** focuses on kinase binding affinities, containing 30,056 interactions with continuous-valued $K_d$ measurements (commonly transformed to $pK_d$).

In our experiments, we treat KIBA scores and $pK_d$ (for Davis) as regression targets. For comparability with prior work, we utilize the already existing dataset splits included with DeepDTA/GraphDTA rather than doing stratified random or scaffold-based splits ourselves.

#### Protein superfamily benchmark

To further evaluate model generalization beyond standard molecular property prediction tasks, we additionally consider an external benchmark consisting of seven protein superfamilies frequently studied in drug–target interaction prediction: enzyme, epigenetic regulator, GPCR, ion channel, kinase, nuclear receptor, and transporter. To avoid potential overlap of drug–target pairs with the Davis and KIBA datasets used in our primary multi-task training pipeline, we evaluate this benchmark separately rather than integrating it into the same training setup.

The datasets were obtained from the benchmark collection introduced by Schulman et al. [43], which aggregates compound–target interaction data derived primarily from the ChEMBL database. The benchmark uses pChEMBL values as continuous bioactivity labels, representing the negative logarithm of molar activity measurements (e.g., $IC_{50}$, $K_i$, or $K_d$). Following the original benchmark protocol, each dataset was randomly split into 80% training and 20% test sets. Table 2 summarizes the dataset sizes for each protein superfamily.

**Table 2:** Summary of external benchmark datasets from the MMAtt-DTA target superfamily benchmark

Dataset	Category	Task type	Labels	Entries
Enzyme	Bioactivity (pChEMBL)	Regression	1	407,967
Epigenetic regulator	Bioactivity (pChEMBL)	Regression	1	104,953
GPCR	Bioactivity (pChEMBL)	Regression	1	188,236
Ion channel	Bioactivity (pChEMBL)	Regression	1	19,573
Kinase	Bioactivity (pChEMBL)	Regression	1	158,038
Nuclear receptor	Bioactivity (pChEMBL)	Regression	1	51,856
Transporter	Bioactivity (pChEMBL)	Regression	1	16,557
Total			7	947,180

“Labels” denotes the number of endpoints/tasks; “Entries” denotes the number of molecule–target pairs

### Model overview

#### Architecture

**Fig. 1:**
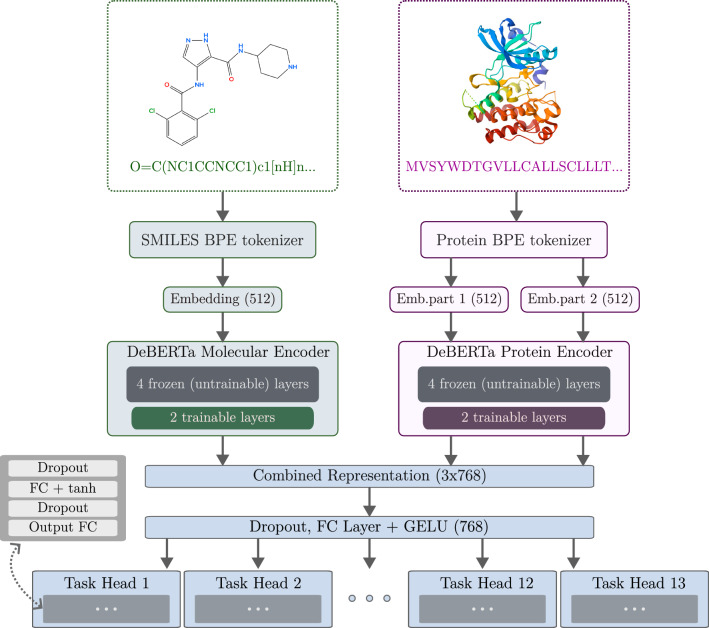
General model architecture and processing pipeline

For simplicity, we start with the general overview of the architecture while the actual pretraining and task-specific training are detailed subsequently. The proposed model builds on a compact DeBERTa backbone [44] and learns shared representations by fusing two lightweight DeBERTa encoders (see Fig. 1). One encoder processes molecular sequences (SMILES), while the other processes protein sequences. Each encoder consists of six transformer layers with 12 attention heads, a hidden size of 768, and a maximum sequence length of 512 tokens, including the special classification token [CLS] and separator token [SEP] markers. We utilize GELU activations and a dropout rate of 0.1, as in the original DeBERTa implementation.

The SMILES encoder is a DeBERTa sequence model. To retain generalizable features learned during pretraining on PubChem, we freeze the first four of its six layers and fine-tune the last two on the downstream property datasets. The protein encoder shares the same architecture and is applied twice to a protein sequence split into two 512-token segments in order to process a token sequence of up to a total of 1024 tokens. Processing shorter segments reduces the quadratic attention cost of long sequences and lowers memory requirements as well as computation time. This strategy allows us to utilize the same protein encoder for both embedding splits, keeping the model size small yet still largely maintaining inter-amino acid interaction capabilities. Both encoders are fine-tuned end-to-end using the same freeze/unfreeze schedule.

The representations from both encoders are then combined and passed to a shared representation layer. For cases where no protein input is provided, the protein-encoder output vector is set to zero. The shared representation layer then provides input to the task-specific output heads. Each output head is implemented as an MLP with 2 fully connected layers with a dropout rate of 0.1 and a tanh activation function, following the default DeBERTa classifier head. The resulting model consists of 96 M parameters, of which 34 M are trainable due to partial encoder freezing.

#### Pretraining

The proposed model follows standard DeBERTa model pretraining adapted to SMILES and protein-sequence inputs.

***SMILES drug encoder pretraining***

1. We obtained the August 2024 PubChem release of SMILES-encoded molecules and sanitized all entries with RDKit. We generated canonical, isomeric SMILES to retain stereochemical information while enforcing a stable representation for attention-based learning. PubChem contains over 117 million compounds; for efficiency, we randomly sampled 50% ($\approx$58 million molecules) for pretraining.
2. We trained a byte-level byte pair encoding (BPE) tokenizer (Hugging Face [45]) on the pretraining corpus with a target vocabulary size of 4,096 and a minimum token frequency of 500. Byte-level BPE iteratively merges the most frequent byte pairs, starting from raw bytes, until the vocabulary limit is reached or no candidate pairs exceed the frequency threshold [46, 47]. The resulting vocabulary contains 2692 tokens and we save the tokenizer for downstream use.
3. Subsequently, we pretrain the SMILES encoder with masked language modeling (MLM) on the tokenized dataset. For each sequence, 15% of the tokens are randomly masked and the model is trained to recover them. The encoder thus learns token dependencies via attention mechanism. This procedure follows an adapted pretraining scheme similar to the original DeBERTa paper. MLM training runs for 3 epochs with a per-GPU batch size of 32 across six RTX 4090 GPUs, a learning rate of $1\times 10^{-4}$, and weight decay of $1\times 10^{-3}$. We use gradient accumulation of 2 steps, yielding an effective batch size of 384 molecules. Sequences are limited to 512 tokens (longer sequences are truncated and shorter ones padded with zeros). We save the pretrained checkpoint for downstream fine-tuning.

***Protein encoder pretraining***

1. We obtained 1.8 million non-redundant protein sequences from a curated dataset [48]. The dataset was compiled from the intersection of the PredictProtein (PP) cache and UniRef50 (release 2019, clustered at 50% sequence identity). Resulting entries were restricted to the standard amino acid alphabet and are later truncated to a maximum input length of 512 tokens for pretraining. The dataset provides train/validation/test splits at the sequence level, of which only the training split was used.
2. We trained a byte-level BPE tokenizer with a maximum vocabulary of 8192 and a minimum token frequency of 10,000. As compared to the molecular tokenizer, we utilize a higher frequency threshold in order to obtain a vocabulary size within our predefined maximum. The higher threshold reduces rare *n*-gram merges, thus keeping the vocabulary compact and focused on the most common sequence patterns. The resulting vocabulary contains 3262 tokens and we save the tokenizer for downstream use.
3. The protein encoder was pretrained with MLM, using the same setup as for the molecular encoder, except for the number of epochs, which we set to 20.

#### Training

After the pretraining phase, both encoders were used to initialize the model weights as described in the previous architecture section. Then, all datasets were canonicalized with RDKit, and isomeric SMILES were enabled for drug compounds. We adopt *dataset-level multi-task learning*, where each benchmark dataset defines one task with its own prediction head and loss function, while the SMILES and protein encoders are shared across all tasks. Thus, DTI data are not pooled into one single task across all source databases. Instead, each DTI benchmark dataset (e.g., Davis and KIBA) is treated as a separate task. Within a given DTI dataset, the model learns a single mapping $(s,p)\!\rightarrow \!y$, where *s* is a SMILES string, *p* is a protein sequence, and *y* is the affinity value, over all ligand–target pairs in that dataset. In contrast, molecule-only property and toxicity datasets define tasks of the form $(s)\!\rightarrow \!y$ or $(s)\!\rightarrow \!\vec {y}$, depending on whether the endpoint is scalar or multi-endpoint.

**Fig. 2:**
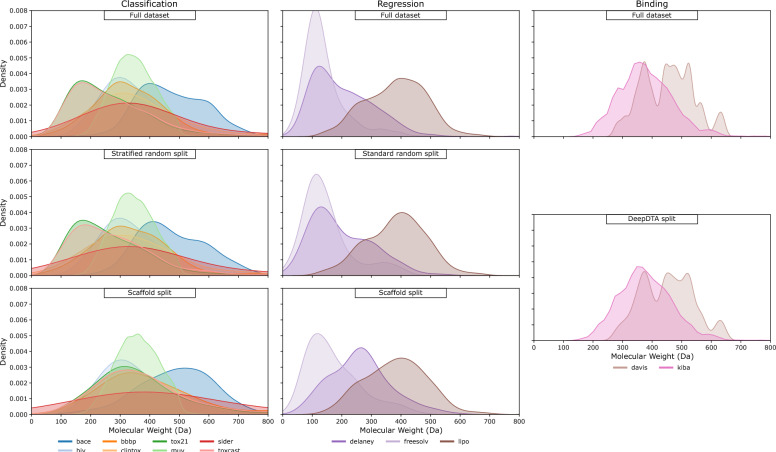
Kernel density estimates of molecular weights for the full dataset (top) and for different split strategies. Randomized test splits preserve the distribution better than scaffold test splits

Train/test splits are defined *per benchmark dataset*, rather than through a single canonical split over the union of all datasets. For classification datasets, we use a stratified random 80/20 split to obtain an even distribution of labels, while the regression datasets are split randomly. The second approach to train/test separation is the scaffold splitting technique, which partitions compounds using structural information [22]. However, as can be seen in Fig. 2, scaffold splitting may introduce additional skew into the data distribution, which is one of the potential issues we must be aware of. Such problems do not appear to arise with randomized splitting techniques.

For molecule-only benchmarks, all labels associated with a given compound are assigned to the same split to avoid within-dataset leakage. For both the KIBA and Davis datasets, we used the same train/test splits as in DeepDTA and GraphDTA. Specifically, each dataset was partitioned into six folds, where one fold was held out as the test set and the remaining five folds were used for training. Because splits are defined per benchmark dataset rather than globally across all tasks, the same compound may occur in different splits across *different* tasks. We adopt this strategy to preserve the standard evaluation protocol of each benchmark, and discuss the resulting cross-task overlap further in the Discussion section.

After loading the datasets, we perform their BPE tokenization with the respective tokenizers. In both cases, if the sequence contains fewer than the required number of tokens, we pad the output with tokens and masks of value 0.

Training uses gradient accumulation of 2 with batch size 32, making the effective batch size 64. We opt for a relatively small batch size to ensure that a model can be experimented and retrained even by a small group of researchers on a single consumer-level GPU (e.g., 16GB mobile GPU training takes 37 h). Other hyperparameters include a learning rate of $5\times 10^{-5}$, weight decay of $5\times 10^{-3}$, FP16 enabled, and 40 epochs. During training, we also utilize a linear warmup of 10% and an 8-bit AdamW optimizer. We then calculate the total number of possible batches and use it to calculate the probability of sampling from a dataset. This sampling probability is then utilized to randomly select a dataset and its batch during training as shown in Table 3.

**Table 3:** Summary of sampling probabilities and combined representations for multitask learning

Dataset	Combined representation	Sampling
ToxCast	SMILES encoder (+ zero padding)	2.71%
SIDER	SMILES encoder (+ zero padding)	0.45%
MUV	SMILES encoder (+ zero padding)	29.31%
Tox21	SMILES encoder (+ zero padding)	2.47%
ClinTox	SMILES encoder (+ zero padding)	0.47%
HIV	SMILES encoder (+ zero padding)	12.95%
BACE	SMILES encoder (+ zero padding)	0.48%
BBBP	SMILES encoder (+ zero padding)	0.64%
Lipo	SMILES encoder (+ zero padding)	1.32%
Delaney	SMILES encoder (+ zero padding)	0.37%
FreeSolv	SMILES encoder (+ zero padding)	0.21%
KIBA	SMILES + protein encoders	38.77%
Davis	SMILES + protein encoders	9.86%
Total	–	100%

After selecting a batch, the corresponding inputs are passed through the available encoders. When no protein sequence is present, the protein encoder is skipped. We then use the [CLS] token from each active encoder to obtain the encoder-wise representations and concatenate them at the feature-fusion stage. For molecule-only datasets, the 768-dimensional SMILES representation is combined with two zero-padded 768-dimensional vectors so that, like DTI samples, all inputs yield a 2304-dimensional fused representation. This fused representation is then passed through a shared representation layer and routed to the prediction head corresponding to the sampled dataset. Thus, via probability-proportional sampling, we achieve multitasking and parameter sharing. We do not merge all datasets into a single rectangular table; instead, training proceeds by interleaving mini-batches drawn from the individual datasets and routing each mini-batch to the corresponding prediction head and loss function. For molecule-only tasks, the protein branch is skipped and the missing protein-dependent representations are implemented as zero-valued placeholder vectors at the feature-fusion stage. For multi-endpoint datasets, the loss is computed only over observed (non-missing) labels via masking.

For ablation, we also train a single-task learning (STL) variant of the model (see Results section) using the same hyperparameters. In this setting, each model is trained on a single dataset with only the corresponding prediction head active, and no interleaving across datasets is performed.

For evaluation on the external benchmark consisting of seven protein superfamilies, a separate multi-task learning (MTL) model was trained on these datasets using the same standard hyperparameters and procedures described above. Dataset sampling and representations are summarized in Supplementary Table S1. Additionally, CheMLT-F was trained individually on each dataset in a single-task learning (STL) setting with the same configuration, except that the training duration was reduced to 25 epochs because each STL model was trained on a single, relatively large external dataset rather than jointly across multiple tasks.

We also evaluated a model initialized from the final checkpoint of the original 13-dataset training. Although a potential overlap between the original training data and the external benchmark may exist, we explored this setup to assess performance when extending the model without retraining the shared components. In this setting, only the task-specific heads were fine-tuned in an STL manner while keeping the encoders and shared representation layers frozen for 40 epochs of training.

#### Loss functions

Under the conditions of multitasking with datasets of different sizes (global context) and possible multi-label classification (local context) with unbalanced dataset labels [22], it is essential to account for potential training skews. We attempt to smooth out this imbalance through the introduction of local and global loss functions.

***Local loss***

For each single dataset, we opt for a context-aware training loss, where classification tasks utilize an adapted focal loss [49], while regression tasks use the mean squared error (MSE). Focal loss offers an improvement over a standard binary cross-entropy (BCE) loss in conditions of varying difficulty in label predictions.

For a binary classification label $y \in [0,1]$ and logit *z*, let $p=\sigma (z)$ be the predicted label probability. The binary cross-entropy (BCE) loss can then be written as

$$\text {BCE}(p,y) = - \, \big [ y \log (p) + (1-y)\log (1-p) \big ].$$

To reduce the contribution of well-classified examples and emphasize harder ones, we utilize the modulating factor

$${\left\{ \begin{array}{ll} (1-p)^{\gamma }, & \text {if } y=1,\\ p^{\gamma }, & \text {if } y=0. \end{array}\right. }$$

which we combine into the unified form

$$w_{\text {focal}}(p,y) = (1-p)^{\gamma } y + p^{\gamma } (1-y),$$

where $\gamma \ge 0$ is the focusing parameter. Using the suggested parameters from the original Focal Loss study, we set $\gamma =2$. Meanwhile, with class weights $\alpha$ derived for each dataset label, the final focal loss becomes

$$\mathcal {L}_{\text {focal}}(p,y) = \alpha \, w_{\text {focal}}(p,y) \, \text {BCE}(p,y).$$

***Global loss*** To account for differences in dataset sizes during multitask training, we scale each task-specific loss by a global dataset-balancing coefficient

$$\beta _i = \sqrt{\frac{\max \pi }{\pi _i}},$$

where $\pi _i$ is the sampling probability of task *i* from Table 3. We utilize the square root to downscale and smooth out potential loss spikes for a more stable gradient descent, which is still aware of the dataset size imbalance. Thus, the final mean-reduced loss for classification task *i* with a batch of size *D* is

$$\mathcal {L}_{i} = \beta _i \,\tfrac{1}{D} \sum _{j=1}^{D} \mathcal {L}_{\text {focal}}(p_j, y_j),$$

while regression tasks use

$$\mathcal {L}_{i} = \beta _i \,\tfrac{1}{D} \sum _{j=1}^{D} (z_j - y_j)^2.$$

In this way, our formulation combines local, task-aware loss functions with global reweighting across datasets of varying sizes.

## Results

### Pretraining dynamics

**Fig. 3:**
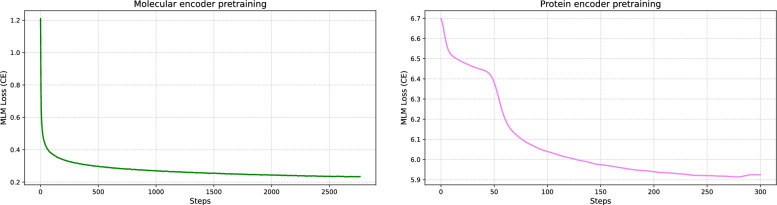
Molecular (left) and protein (right) pretraining MLM loss

Masked-language pretraining converges smoothly for the SMILES encoder (cross-entropy $\sim \!0.2$), whereas the protein encoder exhibits a higher asymptote ($\sim \!5.9$) and a transient saddle, which occurs early during training (Fig. 3). Despite this, downstream DTI results remain competitive, implying that protein features extracted under the current tokenizer are sufficient. Nevertheless, pretraining on a larger protein dataset, improved tokenization or biology-aware masking could reduce pretraining loss and may further benefit DTI (see Discussion section for further details).

### Training results

The results of multitask and single-task CheMLT-F models after 40 epochs of training are shown in Table 4. For CheMLT-F (MTL) and Galactica, each row represents final multitask performance after a single unified training run. All other remaining models are single-task with each training run being represented by a single table cell. Training requires at least 12GB GPU RAM. Inference on the full training set takes 285 s, leading to an average processing time of 1.1 ms/sample when doing batches of 256.

**Table 4:** Performance of models on classification and regression datasets with originally reported values

	Classification (AUC $\uparrow$)								Regression (RMSE $\downarrow$)				
Model	BBBP	ClinTox	HIV	Tox21	SIDER	MUV	ToxCast	BACE	Lipo	Delaney (ESOL)	FreeSolv	KIBA	Davis
CheMLT-F MTL	**0**.**941** $^{a,\dagger }$	0.932$^{a}$	0.824$^{a}$	**0**.**881** $^{a,\dagger }$	0.663$^{a}$	0.831$^{a}$	0.768$^{a}$	0.907$^{a}$	**0**.**528** $^{a,\dagger }$	0.265$^{a}$	**0**.**253** $^{a,\dagger }$	0.376$^{c}$	**0**.**462** $^{c,\S }$
	0.863$^{b}$	0.948$^{b}$	0.789$^{b}$	0.786$^{b}$	0.605$^{b}$	0.776$^{b}$	0.681$^{b}$	0.753$^{b}$	0.619$^{b}$	0.467$^{b}$	0.878$^{b}$	0.376$^{c}$	**0**.**462** $^{c,\S }$
CheMLT-F STL	0.935$^{a}$	0.936$^{a}$	**0**.**852** $^{a,\dagger }$	0.843$^{a}$	0.671$^{a}$	0.818$^{a}$	0.745$^{a}$	**0**.**910** $^{a,\dagger }$	0.534$^{a}$	**0**.**256** $^{a,\dagger }$	0.306$^{a}$	0.386$^{c}$	0.479$^{c}$
	0.870$^{b}$	0.955$^{b}$	0.805$^{b}$	0.758$^{b}$	0.614$^{b}$	0.665$^{b}$	0.679$^{b}$	0.772$^{b}$	**0**.**583** $^{b,\ddagger }$	**0**.**434** $^{b,\ddagger }$	**0**.**694** $^{b,\ddagger }$	0.384$^{c}$	0.471$^{c}$
TrimNet [14]	0.850$^{b}$	**0**.**948** $^{a,\dagger }$	0.804$^{b}$	0.860$^{a}$	0.657$^{a}$	**0**.**851** $^{a,\dagger }$	0.777$^{a}$	0.878$^{b}$	X	X	X	X	X
Dual-view [27]	0.778$^{b}$	**0**.**956** $^{b,\ddagger }$	0.814$^{b}$	0.791$^{b}$	**0**.**698** $^{b,\ddagger }$	X	X	**0**.**894** $^{b,\ddagger }$	X	X	X	X	X
ChemBERTaV1 [11]	0.643$^{b}$	0.733$^{b}$	0.622$^{b}$	NC	X	X	X	X	X	X	X	X	X
ChemBERTaV2 [12]	0.742$^{b}$	0.601$^{b}$	X	NC	X	X	X	0.799$^{b}$	0.744$^{b}$	0.858$^{b}$	X	X	X
Uni-Mol [24]	0.729$^{b}$	0.919$^{b}$	0.808$^{b}$	0.796$^{b}$	0.659$^{b}$	**0**.**821** $^{b,\ddagger }$	0.696$^{b}$	0.857$^{b}$	0.603$^{b}$	0.788$^{b}$	1.480$^{b}$	X	X
Galactica [23]	0.661$^{b}$	0.826$^{b}$	0.745$^{b}$	0.689$^{b}$	0.632$^{b}$	X	X	0.617$^{b}$	X	X	X	X	X
DMPNN [26]	0.913$^{a}$	0.894$^{a}$	0.816$^{a}$	0.845$^{a}$	0.646$^{a}$	NC	0.737$^{a}$	0.878$^{a}$	0.596$^{a}$	0.665$^{a}$	1.167$^{a}$	X	X
	0.888$^{b}$	0.870$^{b}$	0.794$^{b}$	0.791$^{b}$	0.593$^{b}$	NC	0.684$^{b}$	0.838$^{b}$	0.653$^{b}$	0.980$^{b}$	2.177$^{b}$	X	X
GLAM [25]	**0**.**932** $^{b,\ddagger }$	X	X	**0**.**841** $^{b,\ddagger }$	0.659$^{b}$	X	**0**.**744** $^{b,\ddagger }$	0.888$^{b}$	0.596$^{b}$	0.592$^{b}$	1.319$^{b}$	X	X
AttentiveFP [13]	0.920$^{b}$	0.940$^{a}$	**0**.**832** $^{b,\ddagger }$	0.858$^{a}$	0.637$^{a}$	0.843$^{a}$	**0**.**805** $^{a,\dagger }$	0.850$^{b}$	0.578$^{a}$	0.503$^{a}$	0.736$^{a}$	X	X
MoleculeNet [22]	0.729$^{b}$	0.832$^{a}$	0.792$^{b}$	0.829$^{a}$	**0**.**684** $^{a,\dagger }$	0.184$^{a}$	0.742$^{a}$	0.867$^{b}$	0.655$^{a}$	0.580$^{a}$	1.150$^{a}$	X	X
DeepTox [21]	X	X	X	0.846$^{a}$	X	X	X	X	X	X	X	X	X
GraphDTA [15]	X	X	X	X	X	X	X	X	X	X	X	**0**.**373** $^{c,\S }$	0.479$^{c}$
DeepDTA [17]	X	X	X	X	X	X	X	X	X	X	X	0.440$^{c}$	0.511$^{c}$
BALM$^{*}$ [19]	X	X	X	X	X	X	X	X	X	X	X	0.716$^{c}$	0.675$^{c}$
MMatt-DTA$^{\triangle }$ [18]	X	X	X	X	X	X	X	X	X	X	X	X	0.457$^{d}$
DTITR$^{\triangle }$ [20]	X	X	X	X	X	X	X	X	X	X	X	X	**0**.**438** $^{d,\P}$

Split types: a - randomized split, b - scaffold split, c - original GraphDTA split, d - custom DTITR split. NC - not comparable (different metric or task count); X - not tested; † Best result for split a,[‡] Best result for split b,[§] Best result for split c,[¶] Best result for split d. ∗ BALM was fine-tuned from the authors’ pretrained checkpoint with only the projection head unfrozen, using simple hyperparameters without extensive tuning. The Davis and KIBA test subsets were adjusted due to overlap with BindingDB pretraining data. The results reflect this limited setup and may improve with additional tuning, alternative checkpoint selection, or further unfreezing of model parameters. See Supplementary Materials Section 3.1 for details. △ MMAtt-DTA and DTITR are recent attention-based DTI methods with public implementations. Their reported Davis results use the DTITR- defined preprocessing and fold construction, including additional filtering, and are therefore not directly comparable to Davis/KIBA results reported under the DeepDTA/GraphDTA protocol.

**Fig. 4:**
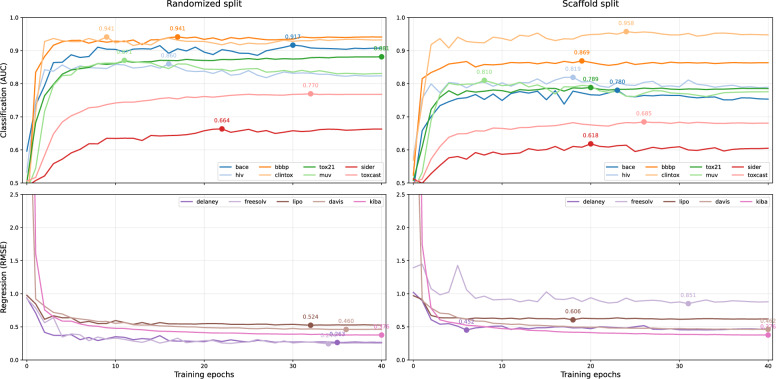
Performance of CheMLT-F (multi-task mode) test-set over epochs. Top: Classification tasks; Bottom: Regression tasks. Left: Randomized split (except Davis and KIBA); Right: Scaffold split (except Davis and KIBA). Numbers indicate best values

Under randomized splits, CheMLT-F reaches AUCs of 0.941 (BBBP) and 0.881 (Tox21), best among entries in Table 4 for the corresponding split type, while remaining competitive on ClinTox (0.932), HIV (0.824), SIDER (0.663), and ToxCast (0.768). Notably, performance is strong despite the breadth of tasks (680+ total endpoints) and the use of a single shared encoder pair with task heads. Figure 4 (left AUC panel) shows early saturation for BBBP and ClinTox, consistent with relatively low label noise and simpler decision boundaries.

On Lipo, Delaney/ESOL, and FreeSolv (RMSE $\downarrow$), CheMLT-F (MTL) achieves 0.528, 0.265, and 0.253, respectively, outperforming single-task graph and transformer baselines reported in Table 4. These results indicate that a compact, shared DeBERTa-based backbone can set strong marks on physicochemical endpoints without task-specific architectures. Figure 4 (left RMSE panel) shows stable epoch-wise improvements with low variance.

For KIBA and Davis (RMSE $\downarrow$), CheMLT-F attains 0.376 and 0.462, respectively. On KIBA, this matches GraphDTA (0.373) within a narrow margin, and surpasses DeepDTA (0.440). On Davis, CheMLT-F improves upon both GraphDTA (0.479) and DeepDTA (0.511). These results suggest that fused sequence encoders offer competitive target-aware representations even without graph inputs.

Under scaffold splits (right panel in Fig. 4), CheMLT-F’s metrics decline across most applicable tasks (e.g., Lipo 0.619, Delaney/ESOL 0.467), reflecting the expected difficulty of scaffold shift. An exception is ClinTox, where AUC improves from 0.932 to 0.948. Single-task counterparts sometimes edge out MTL on regression (e.g., Lipo 0.583, Delaney/ESOL 0.434, FreeSolv 0.694 for STL), suggesting that while shared representations help data efficiency, task-specific adaptation may further aid out-of-scaffold generalization.

Table 4 contrasts CheMLT-F in multitask (MTL) and single-task (STL) modes. On random splits (type a), MTL delivers moderate improvements on several endpoints, including BBBP ($+0.006$ AUC), MUV ($+0.013$), Tox21 ($+0.038$), and ToxCast ($+0.023$), and lowers RMSE on Lipo ($-0.006$), FreeSolv ($-0.053$), and both DTI datasets (KIBA $-0.010$, Davis $-0.017$). Performance is comparable on ClinTox ($-0.004$) and BACE ($-0.003$), and STL holds an edge on HIV ($+0.028$ AUC), SIDER ($+0.008$), and Delaney/ESOL ($-0.009$ RMSE). Under scaffold splits (type b), MTL helps generalization on multi-assay settings - most notably MUV ($+0.111$ AUC) and Tox21 ($+0.028$). However, STL is stronger on several single-endpoint regression tasks (e.g., Lipo $0.583$ vs. $0.619$; ESOL $0.434$ vs. $0.467$; FreeSolv $0.694$ vs. $0.878$). Taken together, these results suggest that shared representations can be useful when tasks are related or label-sparse (multi-assay toxicity/bioactivity), whereas additional STL fine-tuning of an output head is beneficial for scaffold splits in low-sample regression datasets. Additional information regarding BCE and RMSE per-sample error distributions can also be seen in Supplementary Material (Figs. S1-S2 Supplementary Material)

Epoch-wise performance plots of MTL (Fig. 4) show early saturation on BBBP, HIV, MUV, and ClinTox. Interestingly, this effect is not limited to small datasets: both HIV (>40k entries) and MUV (>90k entries) plateau rapidly, reflecting the role of class imbalance, task difficulty and assay noise. Moreover, STL (Figs. S3-S4 Supplementary Material) shows even more prominent early saturation with additional significant overfitting drift on the MUV dataset. Regression tasks, by contrast, improve more gradually across epochs for both MTL and STL. These observations suggest that the current global–local loss approach might benefit from additional automated per-head adjustments (e.g., loss temperature, label smoothing) for additional late-epoch gains.

Using the benchmark introduced by Schulman et al., we evaluated CheMLT-F on seven protein superfamilies: enzyme, epigenetic regulator, GPCR, ion channel, kinase, nuclear receptor, and transporter. This benchmark provides a complementary evaluation setting focused on target-family–specific activity prediction using pChEMBL activity values.

Table 5 summarizes the RMSE results obtained for each protein superfamily. We compare the reported MMAtt-DTA performance with three configurations of CheMLT-F: (i) a 40-epoch fine-tuned CheMLT-F model initialized from the final checkpoint of the main experimental setup; (ii) a fully retrained STL model trained with the standard parameters for 25 epochs; and (iii) a fully retrained MTL model with shared parameters (for details, please see the corresponding Training section). Similar to our previous evaluation on 13 datasets, we report the model performance on the test set after the full training procedure.

As shown in Supplementary Figures S6–S8, adding a task-specific head and fine-tuning it in isolation leads to rapid saturation, with performance plateauing around epoch 20. This aligns with our findings for the BALM model, where we also fine-tuned the model’s final projection layer (Supplementary Figure S5). Retraining the model from scratch proved much more effective, as it allowed us to unfreeze two encoder layers and the shared representation. This pattern is evident in both the STL and MTL full-retraining settings (Supplementary Figures S7 and S8, respectively).

Overall, the retrained MTL CheMLT-F achieved lower RMSE values than MMAtt-DTA for five of the seven protein superfamilies, including enzyme, GPCR, ion channel, kinase, and transporter. The results demonstrate that CheMLT-F’s multi-task training strategy remains effective across diverse protein superfamilies while maintaining competitive performance across heterogeneous bioactivity datasets.

**Table 5:** RMSE comparison across protein families (external benchmark)

Method	Enzyme	Epig. reg.	GPCR	Ion ch.	Kinase	Nucl. rec.	Transporter
MMAtt-DTA	0.509	**0.560**	0.679	0.644	0.625	**0.779**	0.696
CheMLT-F (STL head-tune)	0.690	0.596	0.963	0.966	0.791	0.939	1.042
CheMLT-F (STL retrain)	0.479	0.594	0.614	0.628	0.596	0.800	0.604
CheMLT-F (MTL retrain)	**0.473**	0.582	**0.605**	**0.594**	**0.585**	0.784	**0.584**

## Discussion

Across a wide diversity of tasks, CheMLT-F establishes strong marks on physicochemical regression (best RMSE on Lipo, ESOL, FreeSolv) and maintains competitive classification AUCs. On DTI datasets, the model matches GraphDTA performance on KIBA within a narrow margin and improves on Davis (Table 4). Interestingly, the strongest gains appear on the regression endpoints, suggesting that the shared backbone may capture general physicochemical regularities particularly well. One plausible factor is that these datasets provide continuous targets with clearer signal and less label ambiguity than many classification tasks, which often rely on hard cut-offs to define “active” versus “inactive” compounds. In addition, some classification benchmarks contain sparse or multiple labels per compound, further increasing task difficulty and ambiguity. Exploring these differences, and evaluating models on richer, continuous measures of toxicity or bioactivity rather than binarized or incomplete labels, could clarify why multitask transformers excel on regression tasks and potentially unlock further gains on classification benchmarks.

A related consideration concerns how benchmark splits are defined. Because splits are defined per benchmark dataset, the same compound may occur in different splits across *different* tasks, a known trade-off discussed by Simm et al. [50]. This does not create train–test leakage *within* any individual benchmark, because each benchmark’s test set remains strictly held out from its own training set. However, it can allow shared representations to benefit from exposure to a compound through another task. In principle, global compound-level splitting could eliminate such cross-task overlap [50], but in small, heterogeneous public benchmarks it may lead to highly imbalanced splits and unstable evaluation metrics. Such global splitting strategies may be more appropriate in larger unified datasets or in industrial and federated settings [29, 50].

The results also question the common assumption that explicit graph-based representations are strictly necessary for state-of-the-art molecular prediction: with large-scale pretraining on canonical, isomeric SMILES and representation sharing across related endpoints, compact sequence transformers might be recovering enough substructures and stereochemical regularities to be sufficient for many tasks. That said, our scaffold-split analyses and prior reports [25] suggest graphs may still confer advantages under distribution shift or when geometry and chirality dominate signal. Thus, it is beneficial to view sequence and graph views as complementary rather than competing.

We observe significant asymmetry in MLM behavior of molecular and protein encoders (Fig. 3): SMILES pretraining converges smoothly, whereas protein MLM plateaus higher with an early saddle. This likely reflects (i) longer-range, nonlocal dependencies in proteins (contacts across dozens of residues), (ii) greater sequence heterogeneity and motif diversity, and (iii) truncation into two 512-token segments, which can sever long-range context. Despite this, downstream DTI remains competitive, suggesting the current encoders can already capture useful regularities. Still, there is headroom: protein pretraining typically benefits from larger corpora and biology-aware objectives (e.g., multiple sequence alignment (MSA) and additional structural information)[51–55]; longer-context or efficient-attention encoders could reduce truncation artifacts [56, 57]; and modest tokenizer tuning (e.g., unigram/SentencePiece with subword regularization) may lower perplexity and stabilize learning. Moreover, as demonstrated both by the external benchmark evaluation of our model (Table 5) and by fine-tuning the projection layer of the BALM model (Table 4), attention-based models may quickly plateau at suboptimal performance during fine-tuning when a large portion of their internal parameters remain frozen. Developing methods for determining an appropriate number of tunable parameters could therefore improve optimization in such scenarios. At present, the most straightforward way to achieve better performance is often to retrain the model by unfreezing a subset of encoder layers and fine-tuning them, provided there is no risk of overlap between newly introduced tasks and those used during the original model training.

While CheMLT-F relies on sequence encoders, several complementary avenues could broaden its representational reach. First, adding a lightweight graph branch (e.g., message passing or attentive GNNs) can strengthen local chemistry, stereochemistry, and substructure context that SMILES alone may not be learning optimally–particularly under scaffold shift  [26]. Second, incorporating 3D/energetic signals–learned from efficient conformers (e.g., ETKDG [58]) or auxiliary QM/ML properties (dipoles, partial charges, conformer energies)–can improve noncovalent interaction modeling and target-aware generalization  [59]. Equivariance-aware architectures offer a further path when 3D cues dominate  [60]. Third, expanding data breadth on the protein–ligand side (e.g., PDBbind or CrossDocked) would enrich structure-guided supervision beyond sequence-only corpora  [61, 62]. Furthermore, curated quantum datasets (QM9) can furnish inexpensive auxiliary tasks for physics-informed pretraining  [63]. In practice, we view these additions as modular: a small graph/3D head or auxiliary losses can be fused with our shared backbone without abandoning its simplicity or efficiency.

With additional compute, we would replace heuristic settings with principled hyperparameter search–Bayesian optimization  [64] and multi-fidelity methods such as Hyperband  [65]. It might also be beneficial to refine learning schedules with alternative warmup strategies. We could also scale pretraining (longer runs, span masking, randomized SMILES augmentation  [66, 67]) and tune tokenizers (SentencePiece/WordPiece with subword regularization  [68]) to reduce perplexity and improve transfer. On the modeling side, adaptive lightweight task modules and uncertainty-aware outputs merit evaluation  [69, 70]. For optimization, established multitask balancing schemes–uncertainty weighting, GradNorm, and multi-objective formulations–could improve stability, optionally paired with simple curricula or temperature-based sampling  [71]. Finally, we could broaden evaluation with repeated cross-validation and calibration diagnostics (e.g., expected calibration error) and consider deep ensembles for uncertainty, as well as modest model-size scaling to probe accuracy–compute trade-offs [72, 73].

A natural next step is to replace the shared MLP with bi-directional cross-attention between molecular and protein encoders, enabling explicit alignment of substructures and residues and stronger target-aware conditioning when both modalities are present. Lightweight cross-attention blocks (inserted after encoder outputs) would preserve the simplicity of the backbone, add minimal parameters, and revert seamlessly to molecule-only inference when no protein is provided. In practice, this fusion could be regularized with extra contrastive losses and combined with task-conditioned gating in the heads, offering a clearer path to improved DTI predictions while keeping training and deployment efficient.

## Conclusion

We introduced CheMLT-F, a compact and efficient multitask transformer that unifies drug–target interaction (DTI) prediction with broad molecular property prediction in a single backbone. Across widely used biochemistry benchmarks, CheMLT-F attains performance comparable to, and in several cases exceeding, strong single-task and specialized baselines, while serving 680+ endpoints (toxicities, biochemical inhibitions, physicochemical properties, and protein interactions) from a single unified model. By sharing parameters across heterogeneous tasks, the approach reduces model fragmentation, simplifies deployment, and enables rapid compound screening without the overhead of maintaining multiple task-specific models. The architecture is readily extensible to new datasets and representations, and its modest computational footprint–training on a single GPU–lowers the barrier to adoption in both academic and industrial settings.

Methodologically, we adapt Focal Loss to the multitask regime with global dataset weighting, thus adjusting for class imbalance and uneven label densities across endpoints. This, together with a simple, modular design, yields a practical balance of accuracy, efficiency, and maintainability.

Looking ahead, we see several directions in which this research may be extended: (i) incorporating additional molecular and protein representations (e.g., 3D conformers, pocket-aware or structure-based encodings, richer tokenization schemes); (ii) complexity-aware curricula and dynamic task sampling to further stabilize training across tasks of varying difficulty; and (iii) explainable-AI analyses to examine model rationale and generate mechanistic hypotheses of biochemical relevance. Taken together, CheMLT-F provides a unified, scalable foundation for multi-endpoint biochemical prediction and a pragmatic path toward faster, more transparent decision-making in molecular design.

## Supplementary Information

Supplementary Material 1. Additional supplementary material is available and contains extra scatter plots of averaged error distributions as well as single task CheMLT-F model performance graphs.

## Data Availability

The full source code, pretrained models, and step-by-step scripts to reproduce all experiments are available at **GitHub** (https://github.com/vmun/CheMLT-F). The repository includes preprocessing utilities and the train/test partitions used in the paper (for both random and scaffold splits), as well as model outputs (predictions and logs) supporting the main tables and figures. All datasets used in this study are publicly available. Data for pretraining the molecular encoder was obtained from **PubChem** (https://ftp.ncbi.nlm.nih.gov/pubchem, last accessed 1 March 2026). Protein sequences and metadata were retrieved from **Zenodo** (https://zenodo.org/records/4300971, release September 2020). Drug–target affinity datasets (KIBA and Davis) were obtained from **GraphDTA** (https://github.com/thinng/GraphDTA, last accessed 1 March 2026). Molecular property/toxicity benchmarks (e.g., **BBBP**, **Tox21**, **ESOL**, **FreeSolv**, **Lipophilicity**, etc.) were taken from **DeepChem** (https://deepchem.readthedocs.io/en/latest/api_reference/moleculenet.html, last accessed 1 March 2026). The external protein superfamily benchmark was obtained from **Zenodo** (https://zenodo.org/records/10589696, last accessed 1 March 2026).

## Abbreviations

R&D: Research and development
ADMET: Absorption, distribution, metabolism, excretion and toxicity
STL: Single-task learning
MTL: Multi-task learning
ML: Machine learning
DL: Deep learning
GNN: Graph neural network
SMILES: Simplified molecular input line entry system
DTI: Drug–target interaction
ADR: Adverse drug reaction
GELU: Gaussian error linear unit
MLM: Masked language modelling
MSE: Mean squared error
BCE: Binary cross-entropy
RMSE: Root mean squared error
AUC: Area under the curve
