# Supplementary material for: CheMLT-F: multitask learning in biochemistry through transformer fusion
Source: J Cheminform. 2026 Apr 16;18:69. doi: 10.1186/s13321-026-01199-1 (PMC13217713; doi:10.1186/s13321-026-01199-1)
Supplement: Supplementary file 1 — Supplementary Material 1. Additional supplementary material is available and contains extra scatter plots of averaged error distributions as well as single task CheMLT-F model performance graphs. [file 13321_2026_1199_MOESM1_ESM.pdf]

Supplementary Material for

CheMLT-F: Multitask Learning in Biochemistry through Transformer Fusion

Vladislav Mun and Siamac Fazli

Overview

This document contains supplementary figures, tables, and additional experimental details supporting the main manuscript. All supplementary items are cited in the main text with an additional prefix (Fig. S1, Table S1, etc.).

1 Supplementary Tables

Table S1: Summary of sampling probabilities and combined representations for the MMAtt-DTA target superfamily benchmark

Dataset	Combined Representation	Sampling
Enzyme	SMILES + protein encoders	43.07%
Epigenetic Regulator	SMILES + protein encoders	11.08%
GPCR	SMILES + protein encoders	19.87%
Ion Channel	SMILES + protein encoders	2.07%
Kinase	SMILES + protein encoders	16.69%
Nuclear Receptor	SMILES + protein encoders	5.47%
Transporter	SMILES + protein encoders	1.75%
Total	–	100%

2 Supplementary Figures

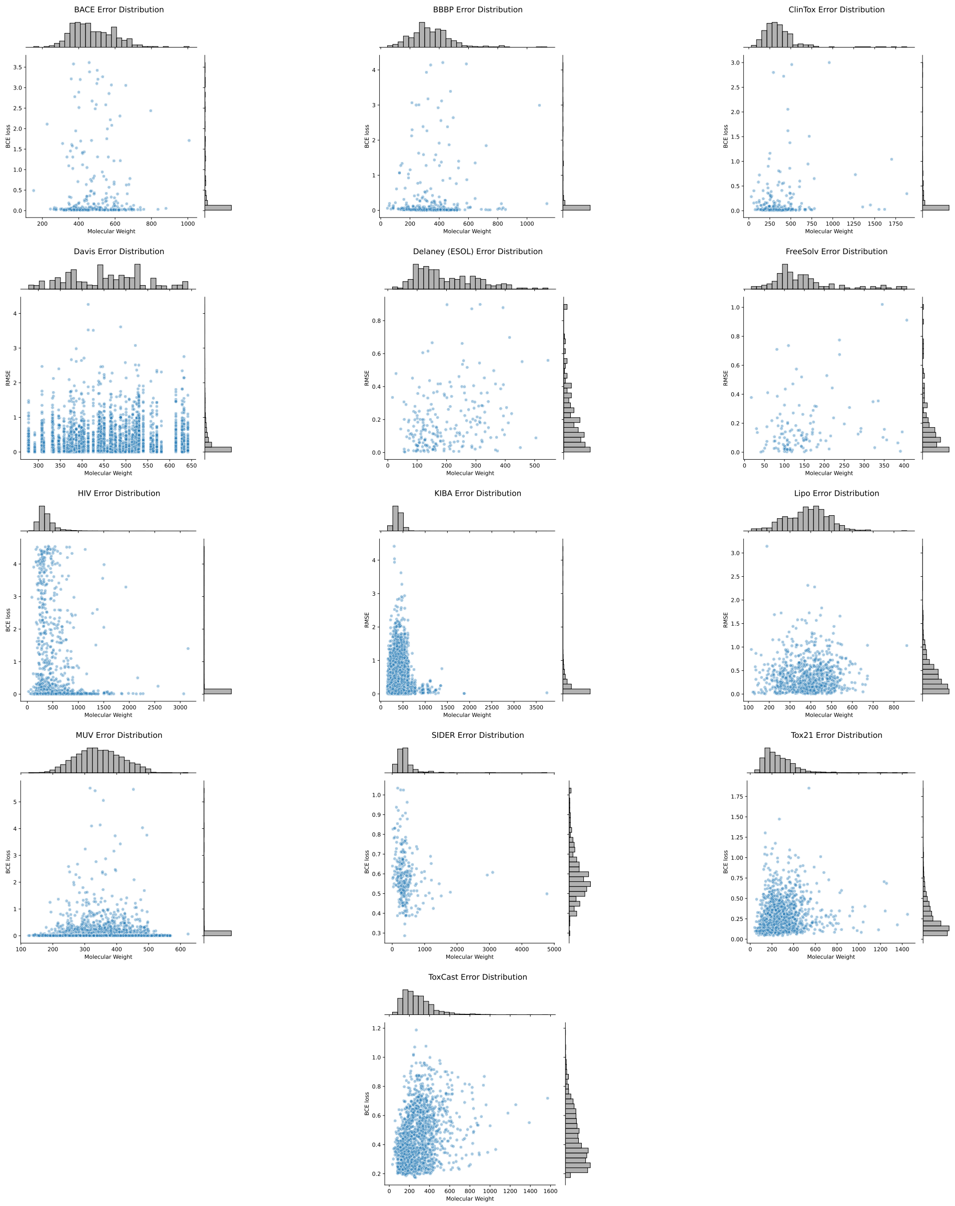

Figure S1: Error distribution plots of CheMLT-F MTL test predictions for 13 benchmark datasets under randomized(a) and custom (c) splits.

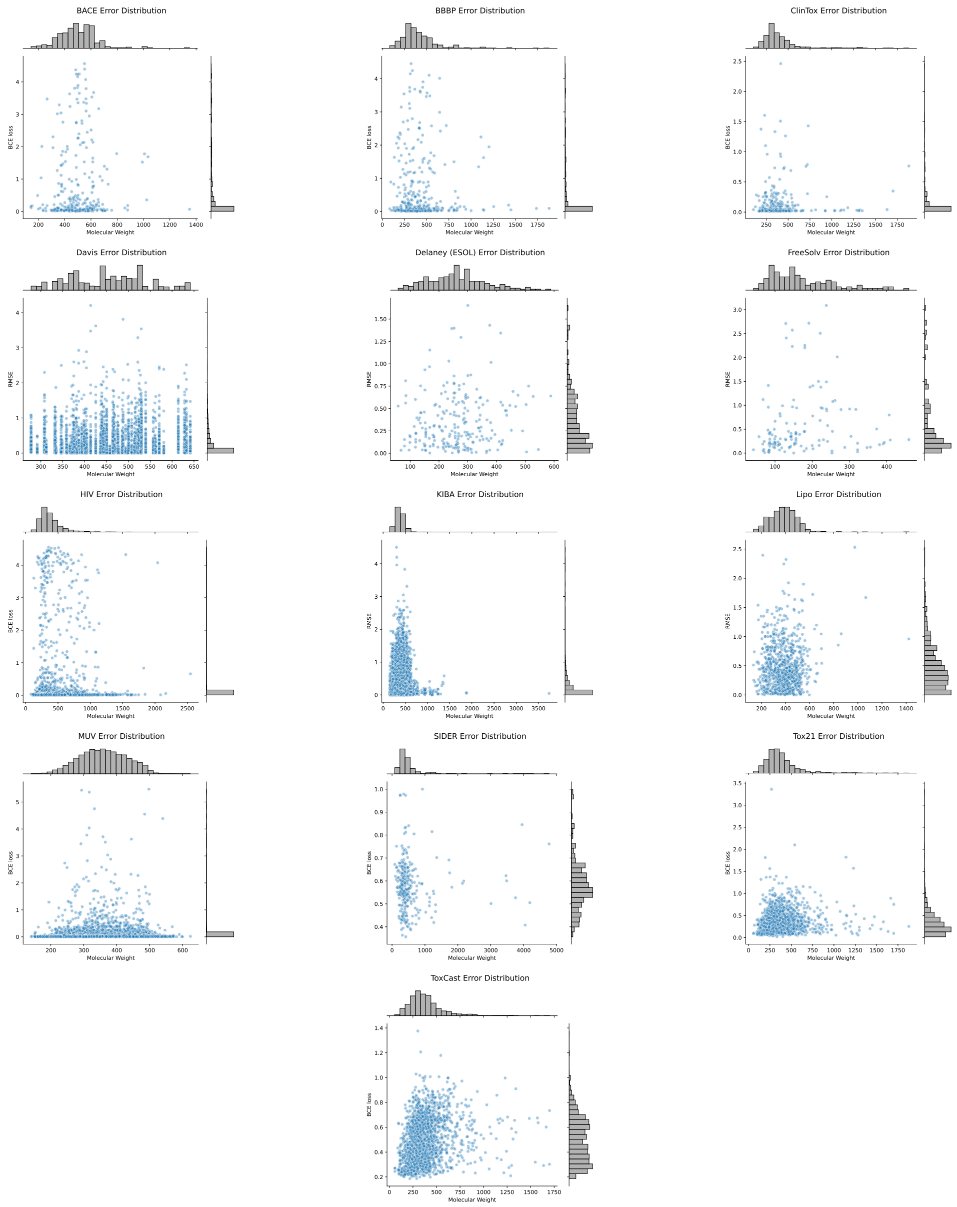

Figure S2: Error distribution plots of CheMLT-F MTL test predictions for 13 benchmark datasets under scaffold(b) and custom (c) splits.

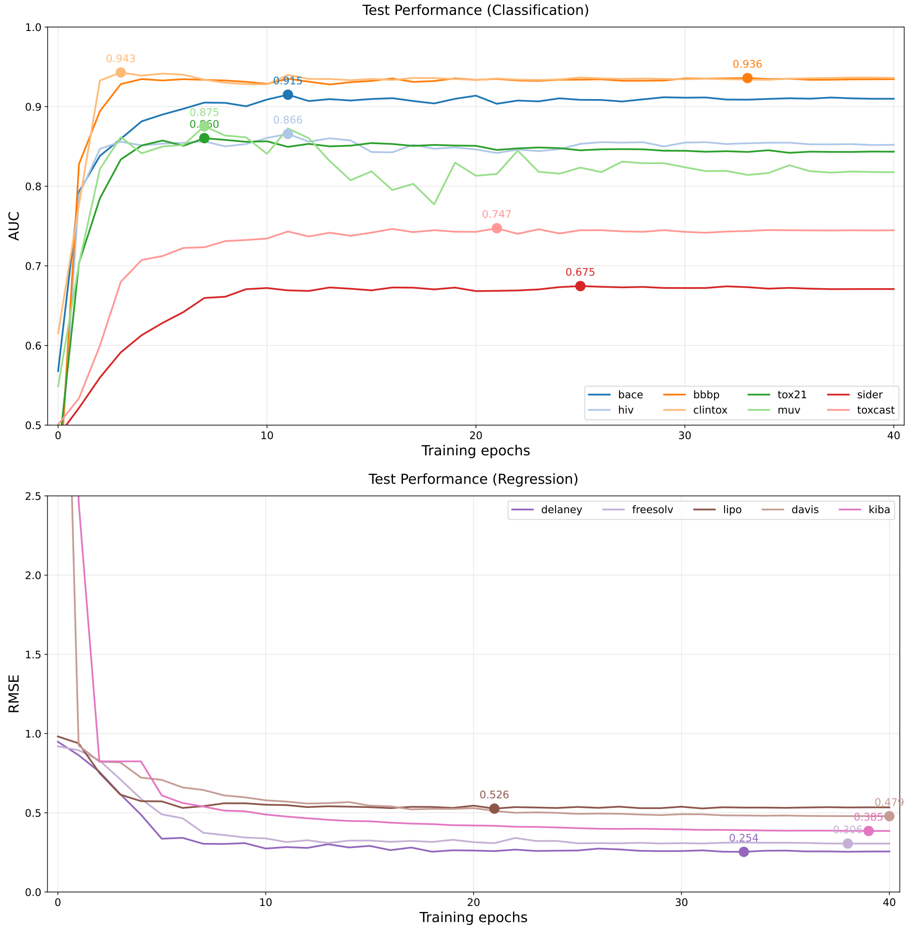

Figure S3: Randomized split test-set AUC (top) and RMSE (bottom) of CheMLT-F in single-task mode over epochs. Numbers indicate best values.

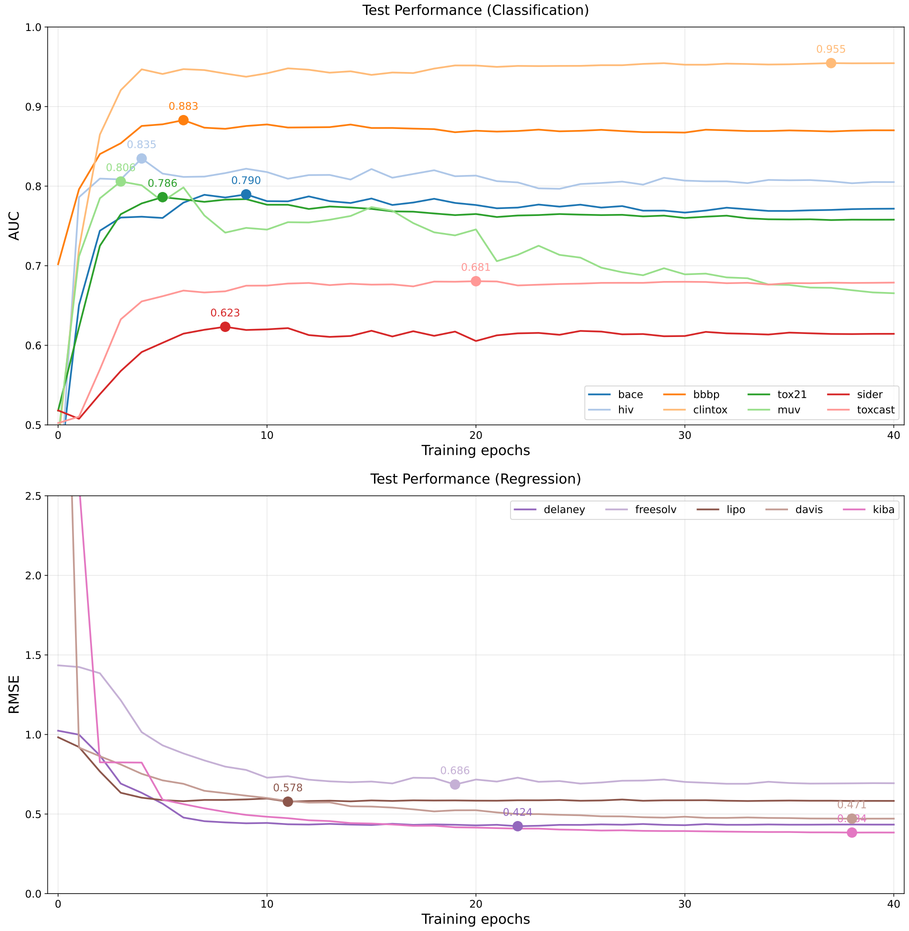

Figure S4: Scaffold split test-set AUC (top) and RMSE (bottom) of CheMLT-F in single-task mode over epochs. Numbers indicate best values.

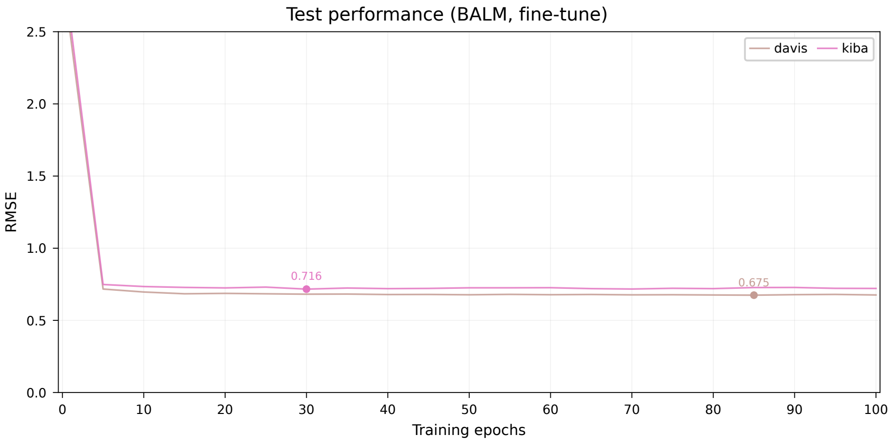

Figure S5: Test performance of BALM fine-tuned projection head

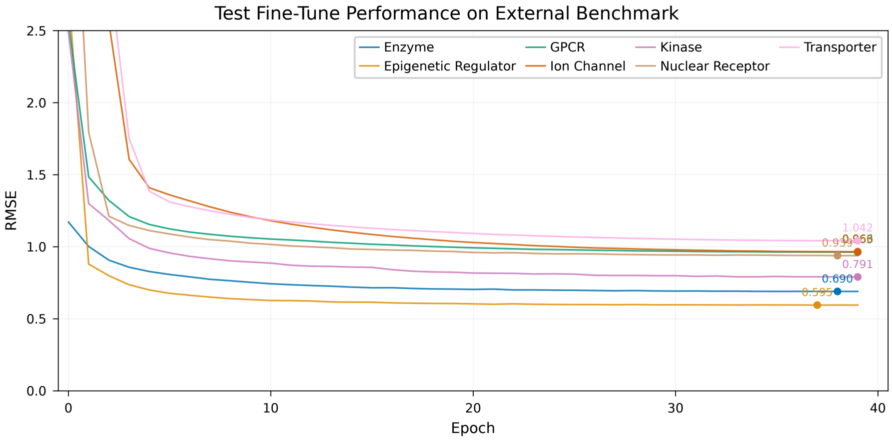

Figure S6: Test performance of the CheMLT-F model initialized from the final checkpoint of the main experimental setup and fine-tuned on the external benchmark

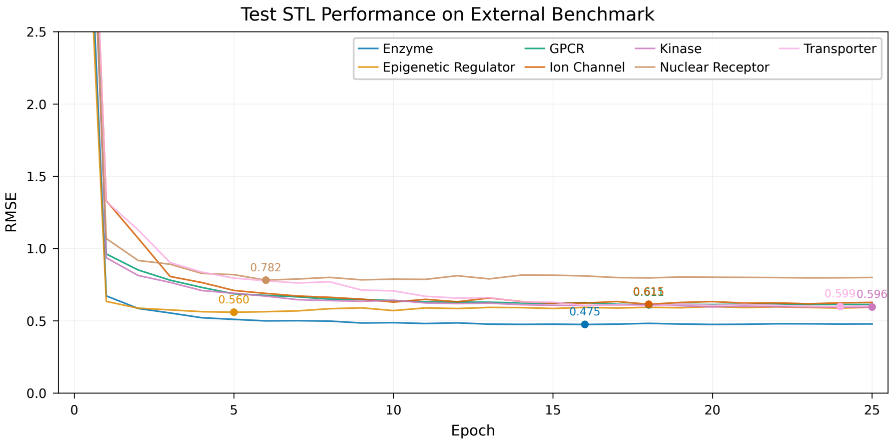

Figure S7: Test performance of CheMLT-F in STL mode, fully retrained on the external benchmark

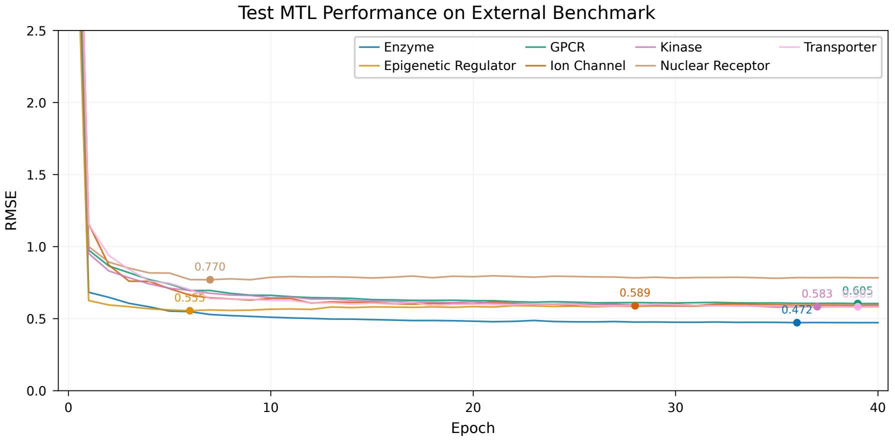

Figure S8: Test performance of CheMLT-F in MTL mode, fully retrained on the external benchmark

3 Additional Experimental Details

3.1 BALM Baseline Implementation

As a recent language-model-based baseline for drug–target interaction (DTI) prediction, we included BALM, proposed in *Learning Binding Affinities via Fine-Tuning of Protein and Ligand Language Models* [1].

For our experiments, BALM was initialized from the BindingDB-pretrained checkpoint released by the original authors and fine-tuned on the Davis and KIBA dataset splits from GraphDTA. The fine-tuning procedure followed the approach suggested in the original work, where all model parameters except for the projection layer are frozen during training.

Training was performed using the AdamW optimizer with a batch size of 64 and a learning rate of 0.001 for 100 epochs, with evaluation every 5 epochs. Prior to training, cosine scaling bounds were updated using the minimum and maximum values of the training set for each dataset separately to ensure correct cosine PEFT calculations.

To avoid potential data leakage, we excluded test entries in Davis and KIBA that overlapped with the BindingDB subset used during BALM pre-training. This substantially reduced the Davis test set (5,010 to 4,472), while KIBA was only minimally affected (19,709 to 19,700).

Training curves for the BALM baseline are shown in Supplementary Fig. S5.

References

[1] R. Gorantla, A. P. Gema, I. X. Yang, Á. Serrano-Morrás, B. Suutari, J. Juárez-Jiménez, and A. S. J. S. Mey. Learning binding affinities via fine-tuning of protein and ligand language models. *Journal of Chemical Information and Modeling*, 65(22):12279–12291, 2025. doi: 10.1021/acs.jcim.5c02063. PMID: 41171175.
